# Understanding the Anion‐Templated, OSDA‐Free, Interzeolite Conversion Synthesis of High Silica Zeolite ZK‐5**

**DOI:** 10.1002/chem.202201689

**Published:** 2022-08-08

**Authors:** Magdalena M. Lozinska, Elliott L. Bruce, James Mattock, Ruxandra G. Chitac, Paul A. Cox, Alessandro Turrina, Paul A. Wright

**Affiliations:** ^1^ EaStCHEM School of Chemistry University of St Andrews Purdie Building, North Haugh St Andrews KY16 9ST UK; ^2^ School of Pharmacy and Biomedical Sciences University of Portsmouth Portsmouth PO1 2DT UK; ^3^ Johnson Matthey Technology Centre Chilton P.O. Box 1, Belasis Avenue Billingham TS23 1LB UK

**Keywords:** anion templating, interzeolite conversion, nitrate, OSDA-free synthesis, zeolite

## Abstract

High silica zeolite ZK‐5 (framework Si/Al=4.8) has been prepared by interzeolite conversion from ultrastable zeolite Y via a co‐templating route using alkali metal cations and nitrate anions but without organic structure directing agents. The mechanism, which involves zeolite framework – alkali metal cation – nitrate anion ordering, has been established by a combination of chemical and thermal analyses, Raman spectroscopy, computational modelling, and X‐ray powder diffraction. Ammonium exchange gives ZK‐5 with occluded ammonium nitrate and subsequent heating gives microporous zeolite ZK‐5.

## Graphical abstract

**High silica forms** of the zeolite ZK‐5 are prepared by an interzeolite conversion using inorganic cations and anions as templates. K^+^ and Cs^+^ cations and NO_3_^−^ anions self‐assemble within its *lta* and *pau* cages with a high degree of space filling and exchange with NH_4_^+^ followed by heating gives a microporous solid. This approach offers an alternative and organic structure directing agent‐free route to directing zeolite synthesis.

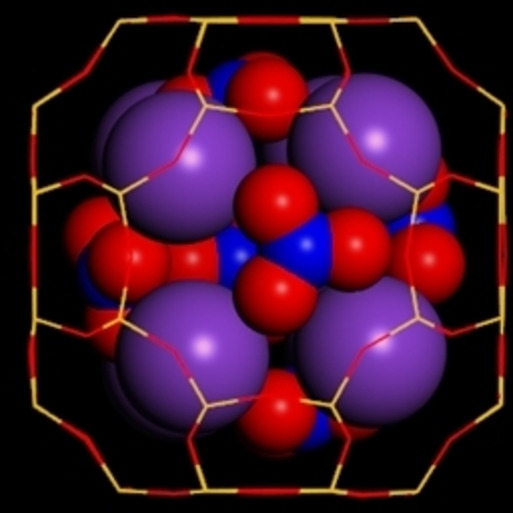

## Introduction

Small pore zeolites are applied as catalysts in small molecule conversions, including the methanol‐to‐olefins reaction and the selective catalytic reduction (SCR) of NO with NH_3_.[^1^, ^2^] For the latter reaction, zeolites SSZ‐13 (topology type CHA) and SSZ‐39 (AEI) are widely used catalysts.[^3^, ^4^, ^5^] Like those zeolites, ZK‐5 (KFI) has a structure built up of *d*6*r* units (double six‐membered rings, 6 tetrahedral cations, six O atoms) with a three‐dimensionally connected pore space comprising cavities with 8R windows and therefore high catalytic potential. In the KFI framework, each *lta* cage opens out onto six *pau* cages through planar 8R windows, while each *pau* cage is linked to two *lta* cages and four *pau* cages (Supporting Information Figure S1). Although the ZK‐5 framework has these attractive structural features for catalysis, the highest Si/Al ratio of as‐prepared ZK‐5 was until relatively recently 4.0, prepared by Chatelain et al. via an 18‐crown‐6 templated synthesis, which restricts its hydrothermal stability.^[6]^ There has therefore been much interest in syntheses of ZK‐5 with higher framework Si/Al ratios to increase their hydrothermal durability. Furthermore, routes have been sought that do not require the use of expensive organic structure directing agents (OSDAs) that must later be removed at environmental cost. Three recent OSDA‐free routes are worthy of note: Kamimura and Endo reported ZK‐5 with Si/Al of 4.0, achieved by seeding; Kim et al. described interzeolite conversion in the presence of excess potassium nitrate (Si/Al=3.9) and most recently Han et al. reported a ZK‐5 prepared from a potassium nitrate rich alkaline gel, with Si/Al of ca. 5.[^7^, ^8^, ^9^] In the last two cases, the materials were active catalysts for SCR when in the Cu,H‐form, with higher Si/Al ZK‐5 showing the better performance after hydrothermal ageing.

As part of a program to understand the synthesis of small pore high silica zeolite catalysts, we are investigating the interzeolite conversion (IZC) route. In IZC, a metastable ‘parent’ zeolite is used as the source of Al and Si for subsequent crystallization of a ‘daughter’ zeolite product through dissolution in alkaline media.^[10]^ The general approach has long been documented – Zones showed it was possible to prepare chabazite SSZ‐13 from ultrastable Y (USY) by this approach, for example,^[11]^ – and in recent years it has been shown to give zeolites with compositions and even structures that are not readily achieved by synthesis from fully amorphous precursors. Important IZC routes starting from USY have been reported for high silica zeolite SSZ‐39 (AEI)[^12^, ^13^, ^14^] and high silica SSZ‐13,^[15]^ the latter making use of a low cost organic structure directing agent (OSDA). IZC has also been applied with inorganic cations^[16]^ and a wide range of OSDAs.^[10]^ Also, many high silica zeolites usually prepared by the use of OSDAs have been prepared by OSDA‐free IZC routes in the presence of seeds.^[17]^ An interesting modification of the synthesis, which interrupts the conversion of USY to Beta in the presence of tetraethylammonium hydroxide, can be tuned to give a series of ‘Interzeolite transformation intermediates’, with little long range order but well‐defined mesoporosity.^[18]^

The mechanism of IZC remains incompletely understood, and is likely to differ between examples.^[19]^ In the majority of cases, the parent and daughter structures have common building units (CBUs), such as small rings and cages, suggesting a pathway where the CBUs prepared in the dissolution step facilitates rapid, directed crystallisation.^[20]^ There is little direct evidence for this, however, and there are cases where there are no CBUs between parent and daughter.^[21]^

The IZC synthesis of ZK‐5, reported by Kim et al.^[8]^ gave a small pore material that could be converted to a small pore catalyst for SCR. Through careful modification of those synthetic conditions, without the use of OSDAs, we have increased the Si/Al ratio of the ZK‐5 product to 4.8. Remarkably, multi‐technique characterisation reveals that under these synthetic conditions the crystallisation of high Si/Al ZK‐5 is directed by both inorganic cations and inorganic anions, and the specificity of this for the KFI framework can be explained in terms of the intrazeolite structural chemistry.

## Results and Discussion

K‐ZK‐5 was synthesised following the interzeolite conversion route of Kim et al.,^[8]^ where the precursor ultrastable zeolite Y (USY), CBV712 (Si/Al=6.2), is first dissolved in an alkaline solution in the presence of an excess of potassium nitrate and then crystallised at 413 K (Table 1). This gives phase pure K‐ZK‐5 with cubic crystals ca. 10 μm in dimension, as previously reported (Figure S2). A synthesis was also followed by PXRD at the lower temperature of 383 K, to monitor the reaction. This showed that the USY precursor had lost all crystallinity before the diffraction peaks of ZK‐5 appear (Figure S3).

**Table 1:** Chemical composition of the synthesis gels and EDS analysis for K,NO_3_‐ZK‐5, K‐ZK‐5(S) and K,Cs,NO_3_‐ZK‐5 samples.

CBV712/CBV720	Chemical composition of the synthesis gels (as molar ratio)								Molar ratio in product ZK‐5 from EDS				
	K^+^	Na^+^	Cs^+^	OH^−^	NO_3_^−^	Al_2_O_3_	SiO_2_	H_2_O	Si/Al	N/Al	K/Al	Cs/Al	(K+Cs)/Al
100/0	15.3	7.2	0.0	1.2	21.3	0.17	2.2	256	4.0	1.1	2.2	0.0	2.2
100/0(NH_4_^+^)	15.3	7.2	0.0	1.2	21.3	0.17	2.2	256	4.0	2.2	0.0	0.0	0.0
70/30	13.8	7.3	1.5	1.2	21.4	0.15	2.2	256	4.7	1.3	2.1	0.8	2.9
50/50	13.8	7.3	1.5	1.2	21.4	0.13	2.3	256	4.8	1.4	1.9	0.7	2.6
40/60	15.3	8.0	1.5	1.4	23.4	0.12	2.3	256	4.4	N.R.	1.8	0.7	2.6
30/70	31.0	14.5	2.0	2.5	45.0	0.11	2.3	256	3.3	N.R.	1.2	0.5	1.7
0/100	38.5	18.0	2.0	3.0	55.5	0.08	2.4	256	3.0	N.R.	1.1	0.5	1.5
K‐ZK‐5(S)^[a]^	0.9	N/A	N/A	0.9	0.02	0.20	2.0	44	4.0	0.0	1.2	0.0	1.2

[a] Additionally Sr(NO_3_)_2_=0.02 and 18‐crown‐6=0.2 were added to the synthesis gel; N.R.– Nitrogen N K*α* emission line not resolved from that of Oxygen K*α*.

Attempts to increase the Si/Al ratio of the product framework were then made by replacing part of the precursor zeolite CBV712 by another USY, CBV720, of higher Si/Al ratio (16.2). However, this resulted in erionite impurity, so some of the KNO_3_ was replaced by CsNO_3_, because Cs^+^ cations favour crystallisation of ZK‐5 (Figure S4).[^22^, ^23^] This gave ZK‐5 up to a 40/60 USY CBV712/CBV720 precursor mix (hereafter described only by the ratio); at CBV720 contents above this, additional alkalinity was required to fully dissolve the USY precursors and obtain phase pure ZK‐5. Full details of the synthesis conditions are given in Section S2 and in Table 1.

**Figure 1:**
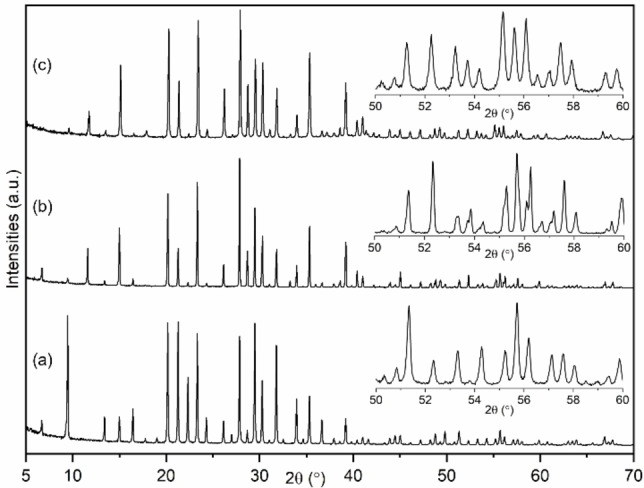
PXRD patterns (Cu K_α1_, λ=1.5406 Å) of representative samples: (a) K,NO_3_‐ZK‐5 (100/0), (b) K,Cs,NO_3_‐ZK‐5 (70/30) and (c) K,Cs,NO_3_‐ZK‐5 (100/0) with, inset, magnified views of 2*θ* range from 50° to 60°.

Powder X‐ray diffraction (PXRD) and scanning electron microscopy (SEM) confirm the preparation of phase pure ZK‐5 in all cases (Figures 1, S2 and S5). Only peaks predicted for the ZK‐5 structure are observed by X‐ray diffraction, although at high 2*θ* the K,Cs 70/30 and 50/50 materials show peak splitting, which we show below is the result of a small distortion from cubic to tetragonal symmetry. The peak intensity differences for the K,Cs‐ZK‐5 materials compared to the K‐ZK‐5 indicate the inclusion of the heavy Cs^+^ cation. SEM reveals the typical cubic morphology of ZK‐5, with some re‐entrant twinning (Figure S2). The crystallite size for the samples prepared at higher alkalinity is smaller (1–2 μm compared to 10 μm) than the others of the series, with surfaces that show more evidence of terraces. Similar changes in morphology of zeolite ZK‐5 were previously attributed to the synthesis conditions, including the Si/Al ratio and the alkalinity of the initial gel.^[7]^

**Figure 2:**
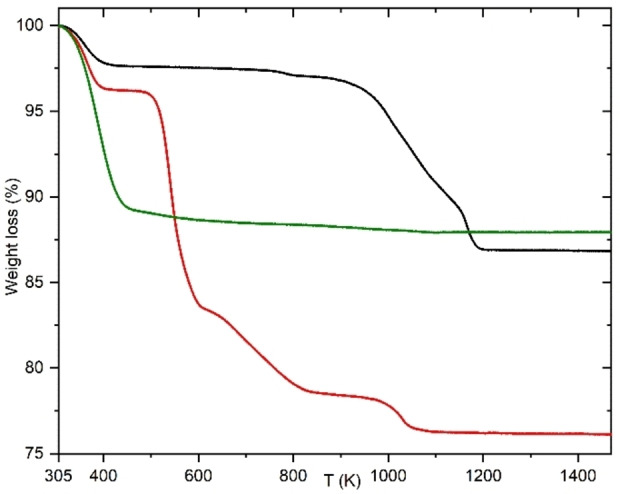
TGA profiles of K,NO_3_‐ZK‐5 (black), NH_4_,NO_3_‐KFI (red) and K‐ZK‐5(S) with 18‐crown‐6 removed by calcination in air at 823 K (green).

Energy dispersive X‐ray spectroscopy (EDS) in the electron microscope shows that the Si/Al reaches a maximum of 4.8 in the mixed precursor, mixed cation materials, while the addition of extra alkalinity results in lower Si/Al framework values (3.3, 3.0) (Table 1). A similar observation was made by Verduijn, who showed that high alkalinity in the synthesis results in a low silica zeolite.^[24]^ Remarkably, these crystals show important differences from ZK‐5 prepared without the addition of high concentrations of KNO_3_. Namely, EDS indicates that (K+Cs)/Al is far in excess of 1 in our materials (Table 1; note K‐ZK‐5(S) is a potassium ZK‐5 prepared via the Chatelain method^[6]^); CO_2_ adsorption by the dehydrated solids (Figure S6) is very low compared to that reported for K‐ZK‐5(S)^[25]^ (at 1 bar, 298 K, <0.8 mmol g^−1^ cf. 4 mmol g^−1^, at 1 bar, 303 K) and TGA shows both very low water contents (ca. 2.5 %) and a sharp weight loss above 900 K (Figures 2 and S7).

Furthermore, careful examination of the PXRD pattern of K,NO_3_‐ZK‐5 revealed that it has a peak intensity distribution distinctly different from that of K‐ZK‐5(S) with a similar Si/Al ratio, indicating the presence of additional framework scattering (Figure S8). This is consistent with the additional extra‐framework cation content observed by EDS (Table 1).

The discrepancy between the Al content of the framework and the K or (K+Cs) content must be due to the presence of anions in extra‐framework positions that balance the charge. In the preparations described here, nitrate or hydroxyl ions were the most plausible candidates, so that additional syntheses and spectroscopic analyses were carried out. In the syntheses, KNO_3_ was replaced by K_2_SO_4_ and K_3_PO_4_ to see if the effect was unique to nitrate ions. It was found that ZK‐5 was formed only if NO_3_^−^ ions are present in addition to K^+^ cations. Sulfate ions, even when present in the synthesis gel, are not included (Figure S9). Additionally, no ZK‐5 is prepared if K^+^ is fully replaced by Na^+^ (Figure S10).

**Figure 3:**
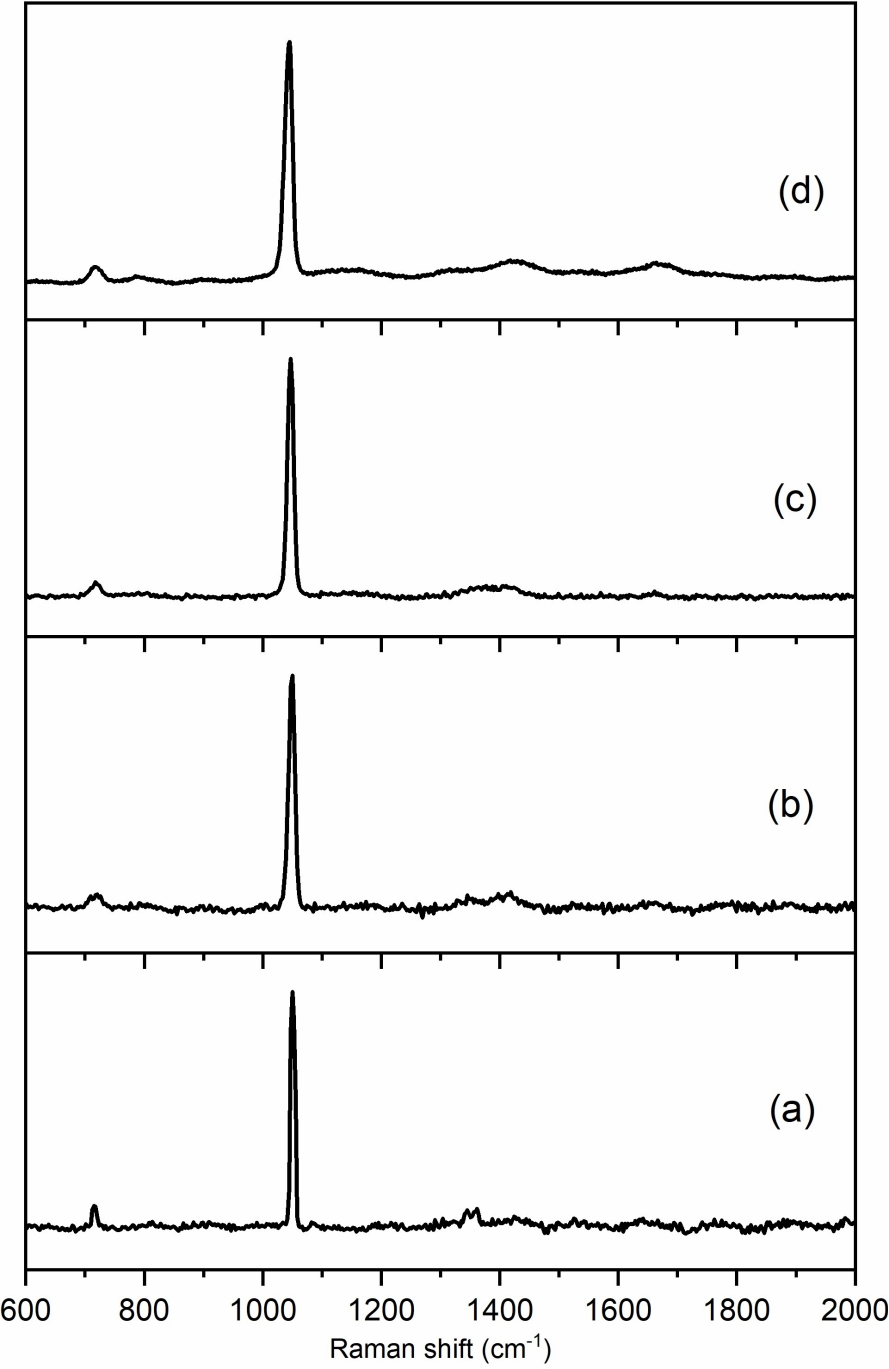
Raman spectra of (a) KNO_3_, (b) K,NO_3_‐ZK‐5, (c) K,Cs,NO_3_‐ZK‐5 (70/30) and (d) NH_4_,NO_3_‐ZK‐5.

In complementary spectroscopic analyses, careful low energy EDS indicates the presence of N emission lines in K,NO_3_‐ZK‐5, with semi‐quantitative analysis indicating a N/Al ratio of ca. 1 (Figure S11 and Table 1). Furthermore, Raman spectroscopy unequivocally indicates the presence of nitrate ions in K,NO_3_‐ZK‐5 and K,Cs,NO_3_‐ZK‐5, even after extensive washing, due to the resonances at 1047–1050 cm^−1^ (*ν*_1,_ strong), 1346–1411 cm^−1^ (*ν*_3_) and 718–721 cm^−1^ (*ν*_4_) (Figure 3).^[26]^

**Figure 4:**
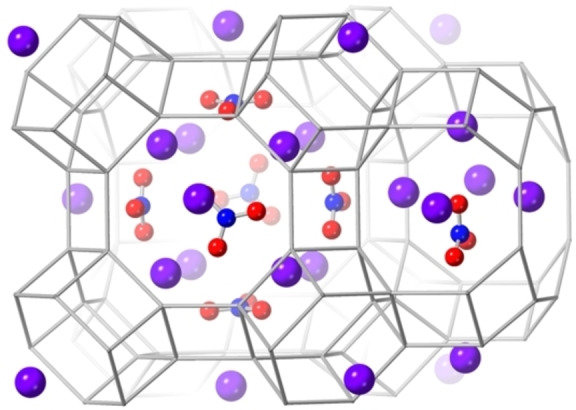
Modelled position of potassium cations and nitrate ions in K,NO_3_‐ZK‐5 (K – purple, O – red and N – blue). Framework O atoms omitted for clarity and T−T linkages represented by grey rods.

These compositional and spectroscopic analyses indicate that nitrate ions, as well as K^+^ cations, are included in the ZK‐5 pores in this synthesis. This accounts for the high observed extra‐framework cation contents (to achieve charge balance), the reduced pore volume available to accommodate H_2_O and CO_2_, and the high temperature weight loss at a temperature at which KNO_3_ decomposes.^[27]^ TG‐MS of K,NO_3_‐ZK‐5 indicates loss of O_2_ and N_2_ during this high temperature mass loss (Figure S12), consistent with KNO_3_ decomposition. Previous cases where nitrate and carbonate anions are included during syntheses of the feldspathoid zeolites sodalite and cancrinite have been reported.[^28^, ^29^] To confirm this hypothesis, an idealised model of K,NO_3_‐ZK‐5 was generated, with some evidence from partial Rietveld refinement that the cation sites I (12), II (12) and III (16) (Figure S1) of the KFI framework are nearly fully occupied. (KNO_3_)_18_K_22_Al_22_Si_74_O_192_ was used as the idealised fully ordered, charge‐balanced starting point (Si/Al=3.4) where all cation sites are occupied and there are 6 NO_3_^−^ anions in each *lta* cage and one NO_3_^−^ anion in each *pau* cage, with the aim of generating starting locations of the anions for subsequent Rietveld refinement. A periodic geometry optimization in *P*1 using interatomic potentials suggested locations for the NO_3_^−^ anions inside the *lta* cage, close to one cation in sites II (S8R) and four in site III (S6R), with close O−K distances of 2.96–3.18 Å (Figure 4).^[30]^ An energy‐minimised configuration was also obtained for the *pau* cage nitrate ion, with O−K distances of 2.85–3.19 Å.

**Figure 5:**
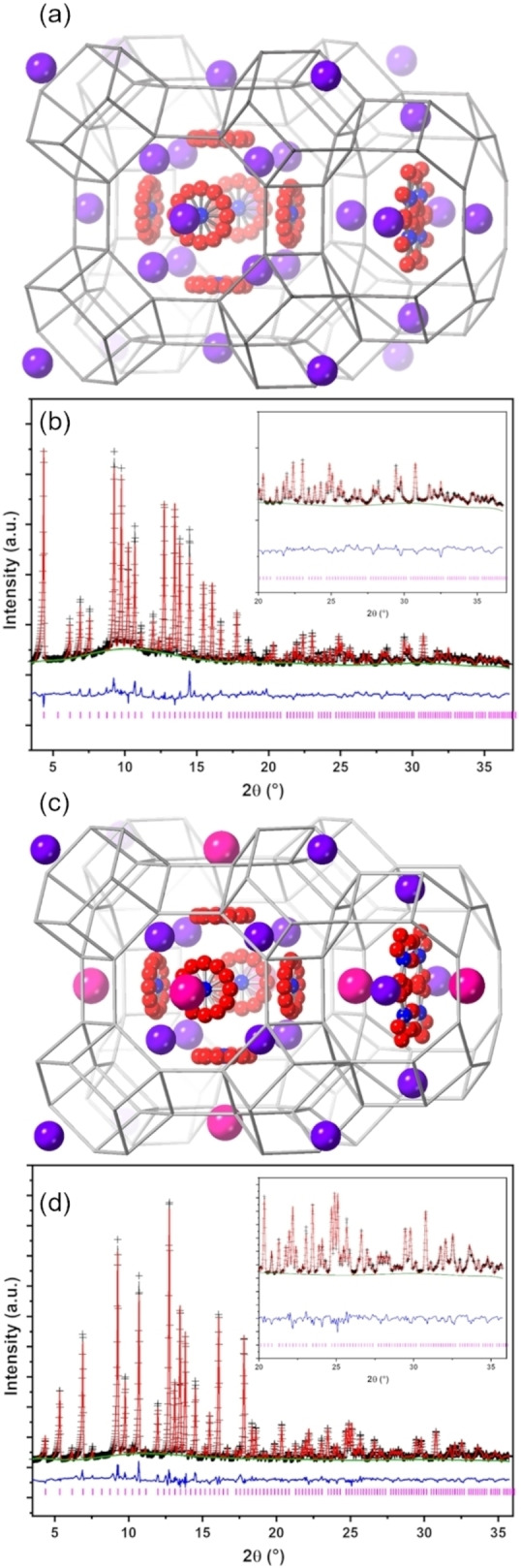
Refined position of cations and nitrate ions in (a) K,NO_3_‐ZK‐5 and (c) K,Cs,NO_3_‐ZK‐5 (70/30) (K – purple, O – red and N – blue. Framework O atoms are omitted for clarity and T−T linkages are represented by grey rods). Rietveld plots of PXRD data (Mo K_α1_, *λ*=0.70926 Å) of dehydrated (b) K,NO_3_‐ZK‐5 and (d) K,Cs,NO_3_‐ZK‐5 (70/30) (Observed – black, calculated – red, difference – blue, phase – pink and background – green).

Rietveld refinement of the structure of dehydrated K,NO_3_‐ZK‐5 using these positions of nitrate ions as a starting point, and refining the cation and anion occupancies and positions, as well as the framework, gave a good fit to the data in $Im\bar{3}m$ , *a*=18.618(1) Å (Figure 5 and Tables S1, S2). This confirmed the high level of K^+^ cations measured by EDS analysis, refining at high fractional occupancy (0.81–0.86) in all available sites in the KFI framework to give a total of 33 K^+^ per unit cell, well in excess of the 19 framework Al cations calculated on the basis of the Si/Al ratio. Approximately 11 NO_3_^−^ anions were refined in *lta* cage sites with an additional 6 NO_3_^−^ anions in *pau* cages. Due to the symmetry, nitrate ions are disordered in the *lta* cages, in a space between K^+^ cations arranged in a tetragonal pyramid, and in the *pau* cages. If the K^+^ cation occupancies from the Rietveld refinement and the framework Al content from EDS are taken to be more accurate compositional measurements than the refined nitrate occupancies, a unit cell composition can be approximated as K_33_(NO_3_)_14_[Al_19_Si_77_O_192_].

A similar structure was obtained by Rietveld refinement against data for K,Cs,NO_3_‐ZK‐5 (70/30), with the difference that Cs^+^ is found to occupy site II exclusively (Figure 5 and Tables S1, S2). These planar S8R sites of the *lta* cages offer the largest minimum M−O distances, suiting the larger Cs^+^ cations and directing the KFI topology. The unit cell composition was established by diffraction as K_24.2_Cs_11_(NO_3_)_17.7_[Al_16.8_Si_79.2_O_192_] with *a*=18.604(1) Å. Additionally, the hydrated K,Cs,NO_3_‐ZK‐5 was analysed structurally to understand the nature of the unit cell distortion. Working from the 2 : 1 intensity of the split peaks (well resolved in the 53–60 °2*θ* range, Figure 1) and removing the cubic symmetry, the structure was refined in the tetragonal symmetry *I*4/*mmm*. (A distortion of the ZK‐5 structure to this tetragonal space group has been observed at high pressure.^[31]^) A good fit to the data was achieved in this space group, with similar cation occupancies to those measured in the dehydrated form (Tables S1, S2 and Figures S13, S14). The large K^+^ and Cs^+^ cations (with additional NO_3_^−^ anions occluded) are not very mobile and do not move upon the adsorption of water, as recently noted by Yang et al.^[32]^ The symmetry change is attributed to the presence of water.

Taken together, these modelled and measured structures confirm that the excess of positive charge on the extra‐framework cations over that required to balance the framework negative charge is compensated by the incorporation of nitrate ions in *lta* and *pau* cages. This explains the high temperature mass loss characteristic of these materials, as well as their low (but non‐zero) uptakes of water and CO_2_: some accessible space remains, despite the presence of the extra‐framework species. The structures suggest that ‘self‐assembly’ of extra‐framework cations and anions inside the ZK‐5 aluminosilicate framework leads to excellent void filling, which is electrostatically driven. It also indicates why sulfate and phosphate ions are not included during synthesis – their tetrahedral geometry and high negative charge would disrupt the K^+^,Cs^+^/NO_3_^−^ ordering.

For these materials to be useful as catalysts, the extra‐framework anionic and cationic species present after synthesis must be removed. The as‐prepared zeolite was therefore ion exchanged by repeated exposure to fresh 3 M NH_4_Cl until no K^+^ remained (Table 1). Raman spectroscopy of this NH_4_,NO_3_‐ZK‐5 (Figures 3d and S15) confirms the presence both of nitrate and ammonium ions (3200 cm^−1^). TGA of NH_4_,NO_3_‐ZK‐5 revealed four mass loss events, 500–610 K (ca. 13 %), 610–840 K (ca. 6 %), 840–1060 K (ca. 2 %) and 1000–1050 K (ca. 2 %) (Figure 2). TG‐MS (Figure S12) indicates the first is loss of physisorbed H_2_O, the second is due to decomposition of ammonium nitrate to N_2_O and H_2_O, the third is from deammoniation and the fourth is due to H_2_O, from dehydroxylation of the H‐form of the zeolite. This confirms the presence of a high level of nitrate ions. Together with the EDS, this indicates that the nitrate ions are not easily lost from the *lta* cages during ion exchange and therefore the ZK‐5 has a much higher cation exchange capacity than would be expected from its Si/Al ratio. Heating the NH_4_,NO_3_‐ZK‐5 at 823 K gave a crystalline H‐ZK‐5 that adsorbed 6 mmol g^−1^ of CO_2_ at 298 K and 10 bar (Figures S6 and S16), indicating a highly porous material. Its uptake at 1 bar, 298 K (3.8 mmol g^−1^), compares well with that of H‐ZK‐5 prepared via the Chatelain method[^8^, ^25^] and it has a very similar PXRD pattern (Figure S16). H‐ZK‐5 with Si/Al=3.9 prepared by IZC and in the presence of nitrates by Kim et al., was found, after Cu^2+^ incorporation, to be an active catalyst for NH_3_ SCR of NO.^[8]^

The synthesis of ‘high silica’ ZK‐5 (i.e. with Si/Al of ca. 5) under OSDA‐free conditions can therefore be achieved by interzeolite conversion in gels rich in both cations and anions. The effect of high ionic strength on crystallisation has been observed previously in zeolite synthesis and explained as a salting out effect,^[33]^ but the inorganic ions play a much more specific and structural role in ZK‐5. We note in passing that that the ‘as‐prepared’ diffraction patterns observed for K,NO_3_‐ZK‐5 are also closely similar to those reported by Han et al.^[9]^ This suggests that the same phenomenon is responsible for crystallization of the ZK‐5 materials they have prepared by nitrate‐rich syntheses and used in the Cu‐form for the NH_3_‐mediated SCR of NO.

The structure of ZK‐5 is well suited to the co‐templating mechanism reported here. K^+^ (and Cs^+^) cations occupy the three available extra‐framework cation sites with very high occupancy, leaving favourable binding sites for the charge‐balancing nitrate anions and resulting in highly efficient void filling. Cs^+^ shows a strong preference for site II, favouring ZK‐5 over competing K‐rich phases such as erionite. The extra‐framework cations balance the charge on the nitrate ions as well as that imparted to the silicate framework by the incorporation of Al. The maximum Si/Al achievable by this route will depend on the number of K cations needed to stabilise the assembly of nitrate ions and the negative charges introduced by the Al substitution.

## Conclusions

The reported routes to ZK‐5 with a Si/Al ratio approaching 5 enable this small pore zeolite to be a stable catalyst. The importance of nitrate ions in the syntheses has been noted previously but here we elucidate the mechanism by which they template the KFI framework, together with K^+^ (and Cs^+^) cations. All available 8R cation sites in the framework are highly occupied by K^+^/Cs^+^ cations, and the size and charge of nitrate ions enable them to pack the pore space efficiently. Furthermore, after ammonium exchange, the occluded ‘salt’ can readily be decomposed to give a crystalline microporous solid acid. This high ionic strength, OSDA‐free synthesis gives a specific route to zeolite ZK‐5 using a combination of K^+^/Cs^+^+NO_3_^−^, but efforts are underway to extend this inorganic cation‐plus‐anion dual templating approach to the synthesis of other zeolites.

## Experimental Section

In a typical synthesis of the CBV712/CBV720=50/50 ZK‐5 sample, 4.6 g of potassium nitrate (Alfa Aesar), 1.68 g of sodium nitrate (Alfa Aesar), 1 g of cesium nitrate (Sigma‐Aldrich) and 4 g of 1 M solution of sodium hydroxide (Alfa Aesar) were dissolved in 11 ml of distilled water under stirring for 2 h. To this solution 0.25 g of CBV712 and 0.25 g of CBV720 were added and it was stirred for additional 2 h. The crystallization was carried out under static conditions in 40 mL stainless steel autoclaves with a Teflon liner for 4 days at 413 K. After reaction, the solid obtained was filtered, washed with distilled water until pH=7 and dried at 373 K overnight. The weight of oven‐dried solid product was 0.31 g.

The crystallinity of all samples was confirmed by laboratory powder X‐ray diffraction (PXRD) using a PANalytical Empyrean diffractometer with a Cu X‐ray tube (Cu K_α1_, 1.5406 Å) and X'celerator RTMS detector. The diffractometer was operated in Bragg‐Brentano reflection geometry, θ‐2θ mode, at room temperature. To determine the structure of dehydrated zeolites, the powders were loaded into 0.7 mm quartz capillaries and dehydrated at 623 K at 5×10^−5^ mbar on a glass vacuum line for 10 h. For the dehydrated samples, a STOE STADIP diffractometer with Mo K_α1_ X‐radiation (0.70926 Å) operated in capillary Debye‐Scherrer mode was used.

SEM imaging was performed on a FEI Scios focused ion beam – scanning electron microscope. Energy dispersive X‐ray spectroscopy (EDS) was performed in a Jeol JSM IT200 scanning electron microscope, equipped with a Jeol DrySD detector. The custom‐made brass stubs with a 2×0.5 mm (diameter×depth) dent (Figure S17 in section S4 in Supporting Information) were prepared to ensure a flat, smooth and compact surface of the samples for EDS measurements. To confirm (K+Cs)/Al values from EDS analysis, two samples, K‐Rho and Cs‐Rho which have been previously extensively characterised,[^34^, ^35^] were used as standards. As a result, instrument K/Al ratios were corrected by dividing by a factor of 1.13 and Cs/Al by 1.17. Counting statistics were good, so all esd's on ratios were ca. 1 %.

Renishaw InVia Qontor confocal Raman microscope was used to characterize the composition of ZK‐5 samples. The Raman spectra were obtained from hydrated samples, using a 532 nm laser radiation. The background was removed in the spectrometer software. The KNO_3_, NH_4_NO_3_ and K_2_SO_4_ purchased from Sigma‐Aldrich were used for comparison.

Thermogravimetric analysis (TGA) of K,NO_3_‐ZK‐5 and NH_4_,NO_3_‐ZK‐5 samples was performed using a Stanton Redcroft STA‐780 instrument with a heating rate of 5 K min^−1^ up to 1073 K in flowing air. The same instrument was used to measure TG‐MS with a heating rate of 5 K min^−1^ up to 1273 K for NH_4_NO_3_‐ZK‐5 and 1623 K for K,NO_3_‐ZK‐5, in flowing argon (Ar). High pressure CO_2_ adsorption isotherms from 0–10 bar at 298 K were measured gravimetrically on a Hiden Intelligent Gravimetric Analyzer (IGA). All samples were activated at 553 K for 10 h prior to measurements. The mass change for each adsorption/desorption step was followed, and a final reading was taken when it had reached 98 % of the asymptotic equilibrium value or after 90 min, whichever was shorter.

The crystal structures were determined by Rietveld refinement against the PXRD data using TOPAS Academic software.^[36]^ The starting framework model for K‐ZK‐5 was adapted from the literature with the unit cell modified to that derived from the diffraction patterns.^[34]^ *I*4/*mmm* symmetry was chosen for the hydrated K,Cs,NO_3_‐ZK‐5 material as its PXRD pattern showed additional peaks consistent with a tetragonal unit cell. *I*4/*mmm* is the only tetragonal daughter of the *Im*‐3 *m* space group and gave a reasonably good fit to the data. Three starting extra framework cation sites were taken from the literature models and their fractional occupancies and atomic coordinates were refined.[^24^, ^34^, ^37^] The position of nitrate ions was obtained as described below and in the Results and Discussion. The framework atomic positions were initially refined with geometric restraints on T−O (T=Si or Al; 1.64±0.02 Å) and O−O (2.65±0.02 Å) distances to maintain regular tetrahedral coordination. Atomic scattering factors were used for all atoms. The peaks were fitted with a Pseudo‐Voigt profile and backgrounds were fitted by 9–11 term shifted Chebyshev function. The crystallographic data for all ZK‐5 structures is given in the Supporting Information and cif files.

In order to assist with resolving the location of the potassium and nitrate ions within the KFI unit cell, a periodic geometry optimisation was performed using the Forcite module within the Materials Studio program.^[38]^ The COMPASSII forcefield was used with charges calculated by the Charge Equilibration method.^[39]^ A fully siliceous model was used for the framework. In order to balance the charge in the system without defining Al positions, a −22 charge was applied across the SiO_2_ framework to counterbalance the charges of the potassium and nitrate ions. The smart algorithm was used to achieve optimisation. An atom‐based summation method was used for both the electrostatic and van der Waals energy contributions. All of the atoms were free to move during the optimisation and no symmetry constraints were applied. The cif file of the energy‐minimised structure in *P*1 is deposited separately.

## Conflict of interest

The authors declare no conflict of interest.

1

## Supporting information

As a service to our authors and readers, this journal provides supporting information supplied by the authors. Such materials are peer reviewed and may be re‐organized for online delivery, but are not copy‐edited or typeset. Technical support issues arising from supporting information (other than missing files) should be addressed to the authors.

Supporting Information

## Data Availability

The data that support the findings of this study are openly available in PURE at https://doi.org/10.17630/c21b9494‐2167‐4492‐b50f‐01c917be928c, reference number 279615097.
