# Supplementary material for: Understanding the Anion‐Templated, OSDA‐Free, Interzeolite Conversion Synthesis of High Silica Zeolite ZK‐5
Source: Chemistry. 2022 Aug 8;28(56):e202201689. doi: 10.1002/chem.202201689 (PMC9804891; doi:10.1002/chem.202201689)
Supplement: Supplementary file 1 — Supporting Information [file CHEM-28-0-s001.pdf]

Chemistry–A European Journal

Supporting Information

Understanding the Anion-Templated, OSDA-Free, Interzeolite Conversion Synthesis of High Silica Zeolite ZK-5

Magdalena M. Lozinska,* Elliott L. Bruce, James Mattock, Ruxandra G. Chitac, Paul A. Cox, Alessandro Turrina, and Paul A. Wright*

S1. Structure of KFI

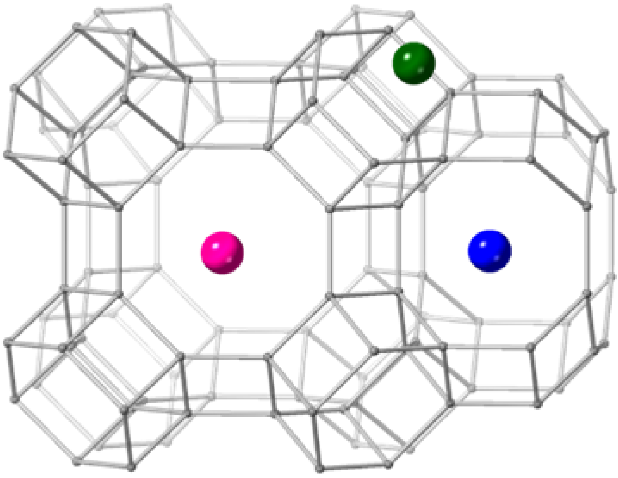

Figure S1. Zeolite KFI framework including the three main cation sites: S8R of the *pau* cage (site I; blue sphere), S8R of the *lta* cage (site II; pink sphere) and S6R of the *lta* cage (site III; green sphere). 6R and 8R refer to the size of the ring, which contains 6 or 8 tetrahedral Si or Al atoms and 6 or 8 O atoms, respectively. Framework O atoms are omitted for clarity and T–T linkages are represented by grey rods.

S2. Synthesis and ion exchange of zeolite ZK-5

The zeolites K,NO_3-ZK-5 and K-ZK-5(S) were synthesised using previously reported procedures.[1,2] The synthesis of K,NO_3-ZK-5 was modified to produce a series of K,Cs,NO_3-ZK-5 samples (see Table 1 in the Manuscript for compositions of the synthesis gels). The starting mixture was prepared by dissolving potassium nitrate (45-127 mmol; Alfa Aesar) and sodium nitrate (18-49 mmol; Alfa Aesar) in distilled water (11 ml). For K,Cs,NO_3-ZK-5 samples, a required amount of KNO_3 was substituted with $CsNO_3$ (5.0-6.5 mmol; Sigma-Aldrich). 1 M solution of sodium hydroxide (3.9-9.8 mmol; Alfa Aesar) followed by zeolites USY, CBV712 or/and CBV720 (0.5 g of zeolite USY in total; oxide and other ratios given in Table 1 in the Manuscript) were added and the mixture stirred until homogeneous. Commercial ultrastable USY zeolites with Si/Al ratio of 6.2 and 16.2 for CBV712 and CBV720, respectively, were obtained from Johnson Matthey. The crystallization was carried out under static conditions in 40 mL stainless steel autoclaves with a Teflon liner for 4 days at 413 K. After reaction, the solid obtained was filtered, washed with distilled water then dried at 373 K overnight.

For zeolite K-ZK-5(S), a solution A was prepared by dissolving aluminium isopropoxide (7 mmol; Sigma-Aldrich) and potassium hydroxide (18 mmol; Alfa Aesar) in distilled water (4 ml). Solution B was prepared by dissolving 18-crown-6 (3.4 mmol; Sigma-Aldrich) and strontium nitrate (0.3 mmol; Alfa Aesar) in distilled water (6 ml) followed by addition of Ludox-AS-40 (83 mmol of SiO_2; Sigma-Aldrich). Solutions A and B were combined, and the mixture stirred until homogeneous. The crystallization was carried out under static conditions in 40 mL stainless steel autoclaves with a Teflon liner for 5 days at 423 K. After reaction, the solid obtained was filtered, washed with distilled water then dried at 373 K overnight.

S3. Characterisation Results

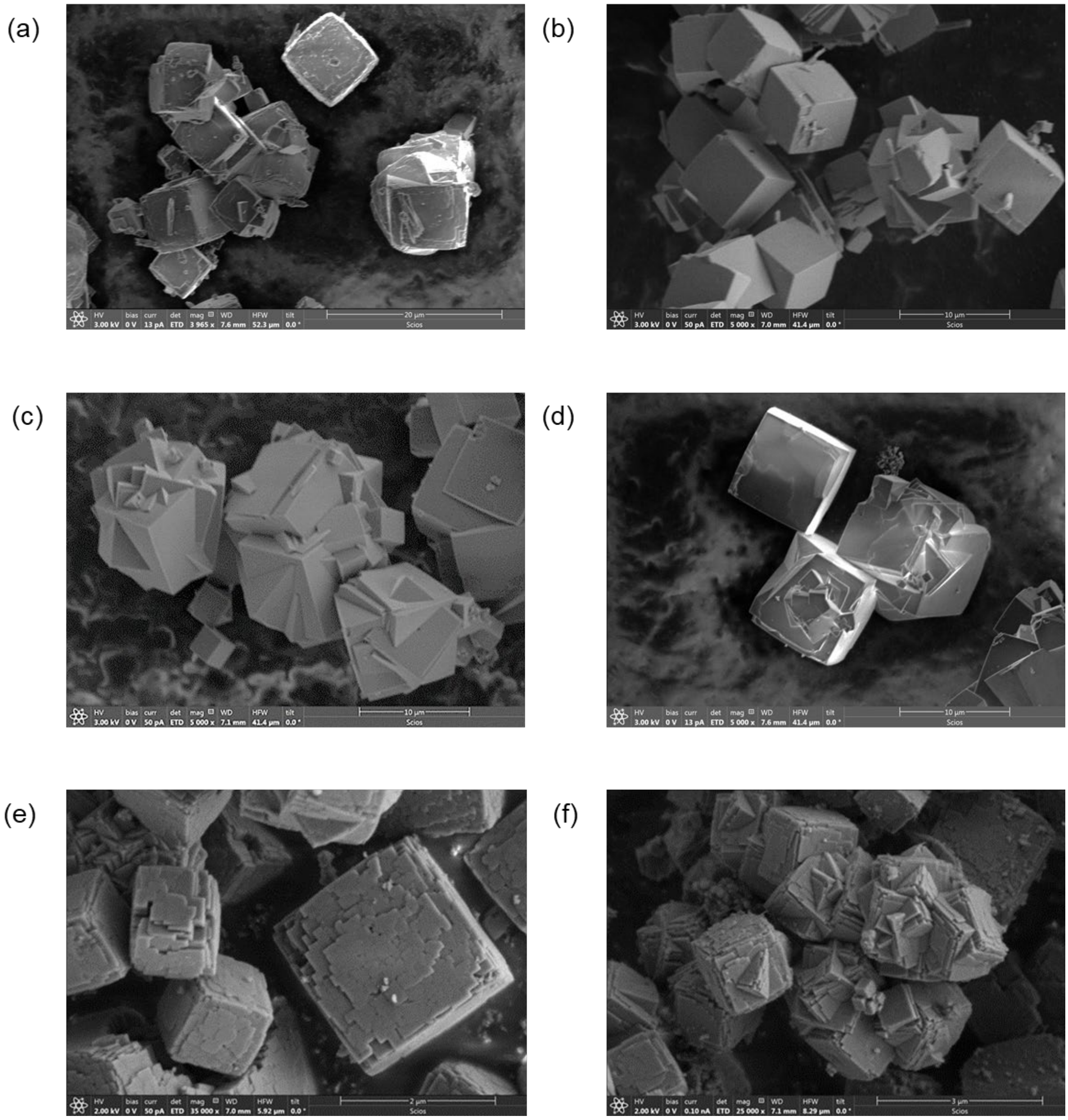

Figure S2. SEM images of K,NO_3-ZK-5 and K,Cs,NO_3-ZK-5 samples synthesised from CBV712/CBV720 ratios of (a) 100/0, (b) 70/30, (c) 50/50, (d) 40/60, (e) 30/70 and (f) 0/100. Compositions given in Table 1 of the paper.

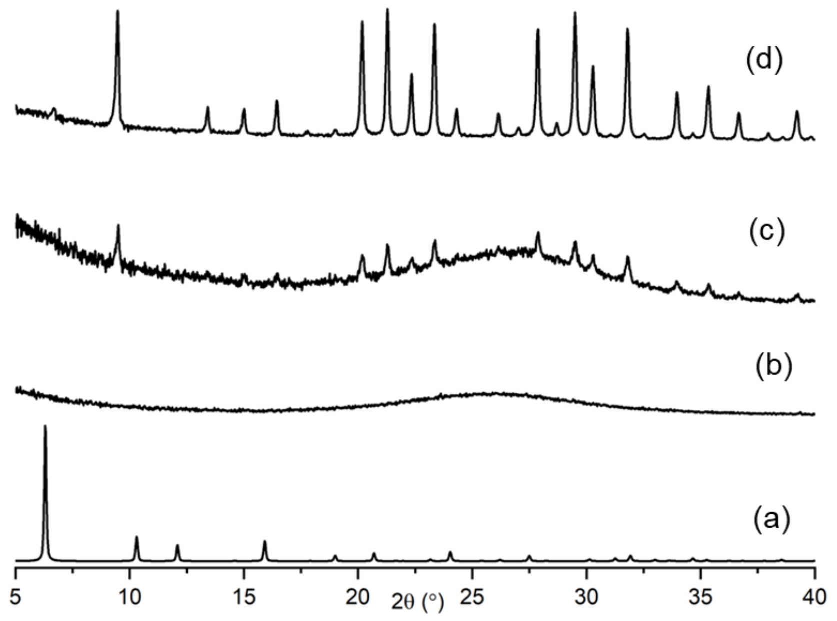

Figure S3. (a) Simulated powder X-ray diffraction pattern of the zeolite Y framework,[3] compared with experimental PXRD patterns of separated solid product at different times of heating the synthesis gel at 383 K. (b) 1 day, (c) 2 days and (d) 4 days.

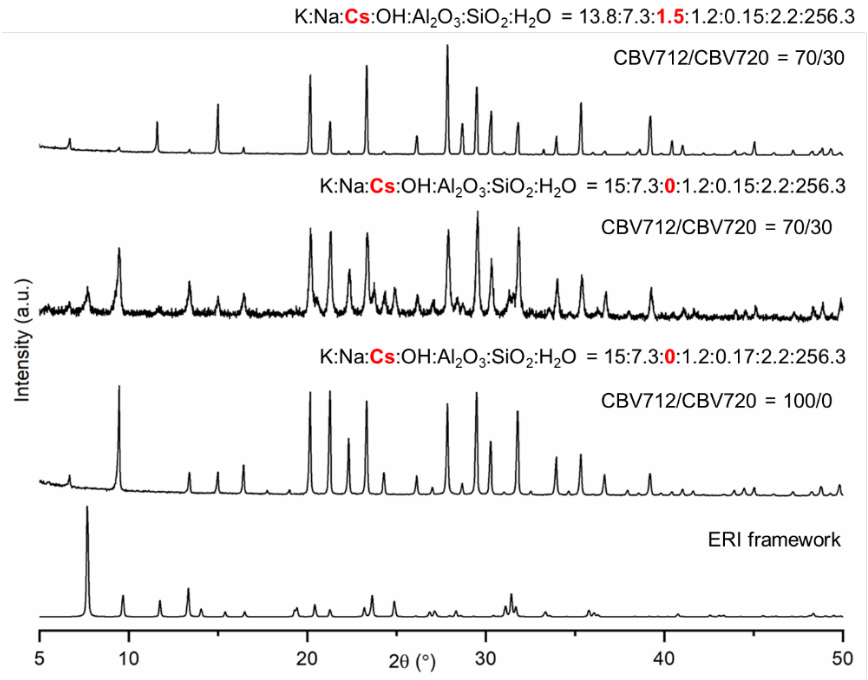

Figure S4. Simulated PXRD of SiO_2(ERI)[3] and measured patterns of K,NO_3-ZK-5 synthesised from a ratio of CBV712/CBV720 = 100/0 and 70/30 with starting gel compositions showing importance of Cs^+ cations in eliminating ERI impurities.

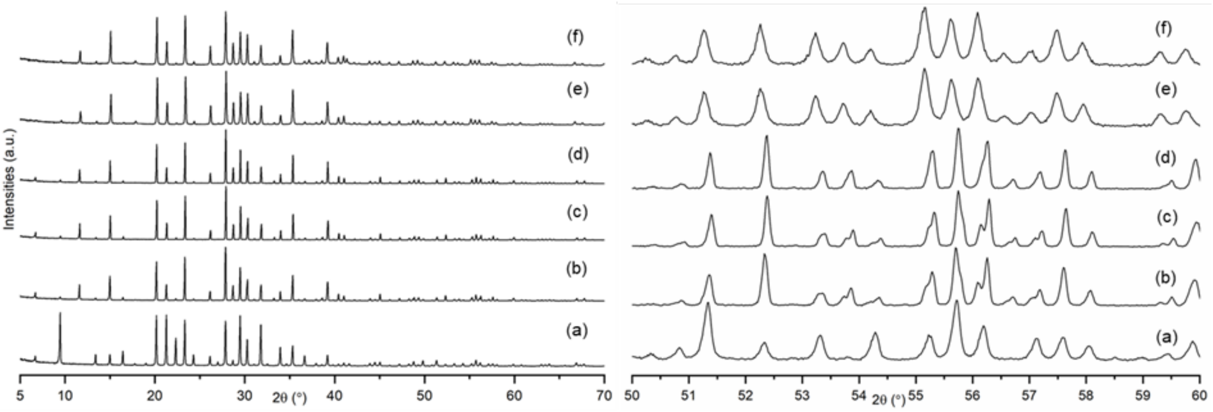

Figure S5. (Left) PXRD patterns of K,NO_3-ZK-5 and K,Cs,NO_3-ZK-5 samples synthesised from CBV712/CBV720 ratios of (a) 100/0, (b) 70/30, (c) 50/50, (d) 40/60, (e) 30/70 and (f) 0/100. (Right) Magnified views of 2θ range from 50º to 60°. See Table 1 for details.

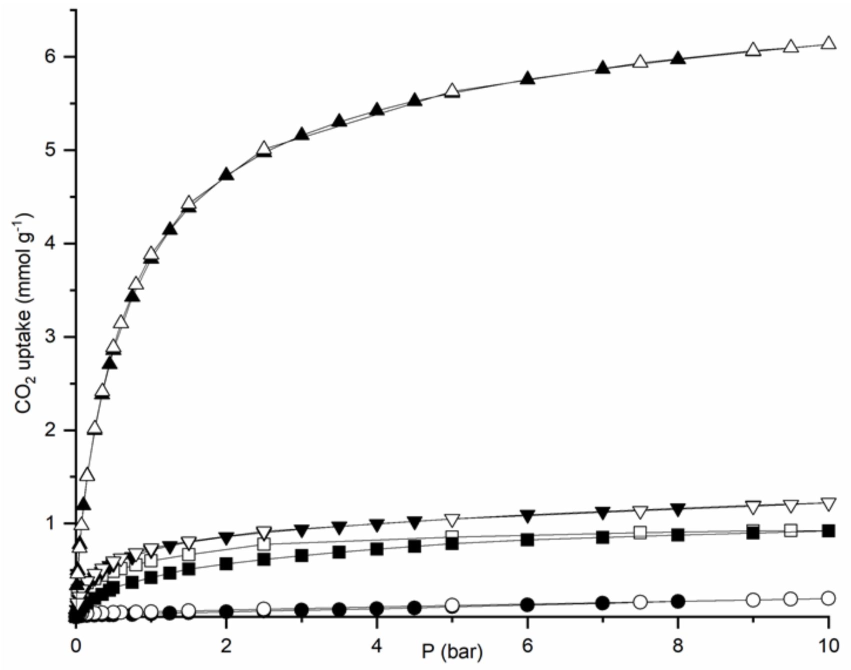

Figure S6. CO_2 adsorption at 298 K on K,NO_3-ZK-5 (▼), K,Cs,NO_3-ZK-5 (CBV712/CBV720 = 70/30) (●),K,Cs,NO_3-ZK-5 (CBV712/CBV720 = 30/70) (■) and H-ZK-5 prepared by heating NH_4,NO_3-ZK-5 in flowing oxygen at 823 K for 12 h (▲). Adsorption, closed symbols; desorption, open symbols.

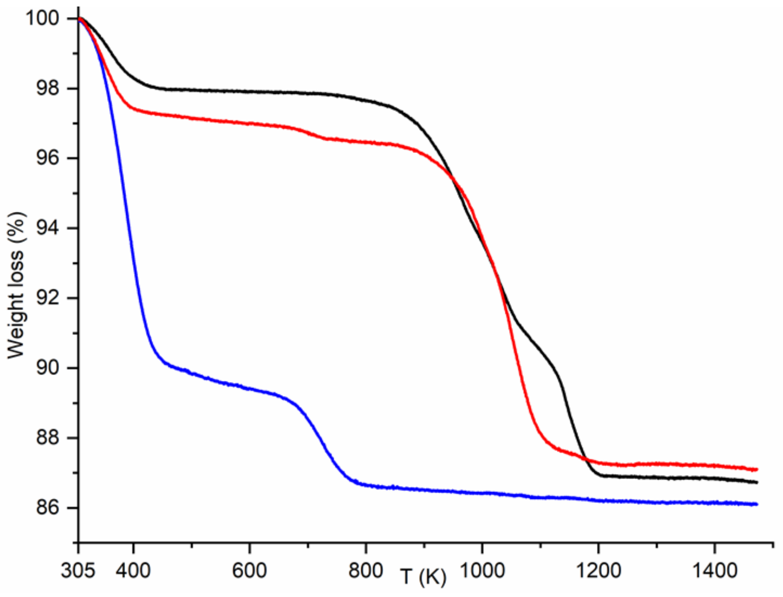

Figure S7. TGA profiles of K,Cs,NO_3-ZK-5 (black; 70/30), K,Cs,NO_3-ZK-5 (red; 0/100) and K-ZK-5(S) (blue; as-prepared, with 18-crown-6 template included).

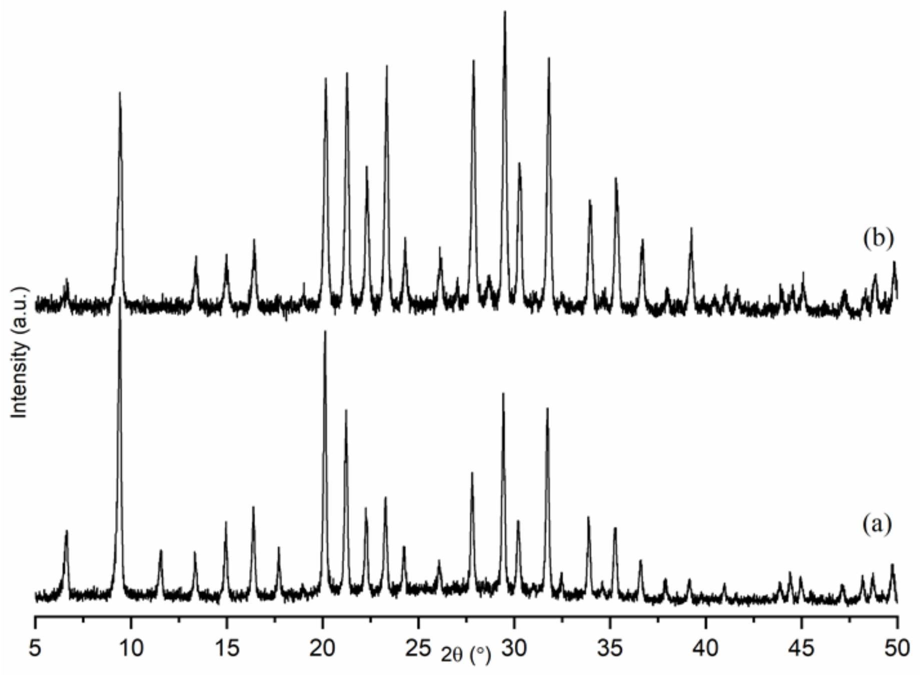

Figure S8. PXRD patterns of as-prepared (a) K-ZK-5(S) and (b) K,NO_3-ZK-5 samples.

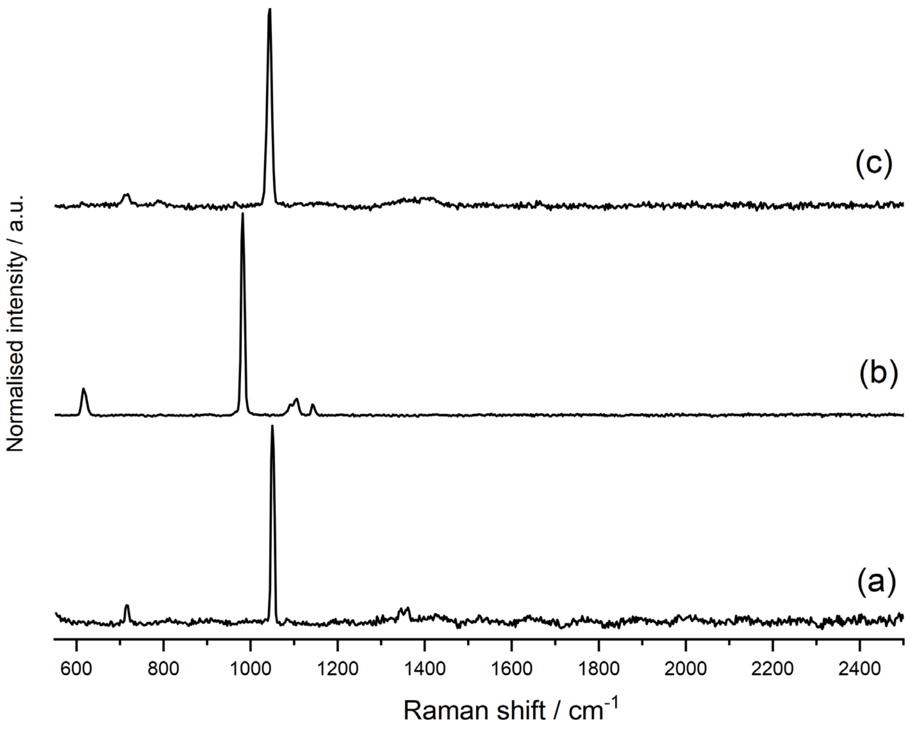

Figure S9. Raman spectra of (a) KNO_3 standard, (b) K_2SO_4 standard and (c) K,NO_3-ZK-5 synthesised using KNO_3 + Na_2SO_4 in starting gel.

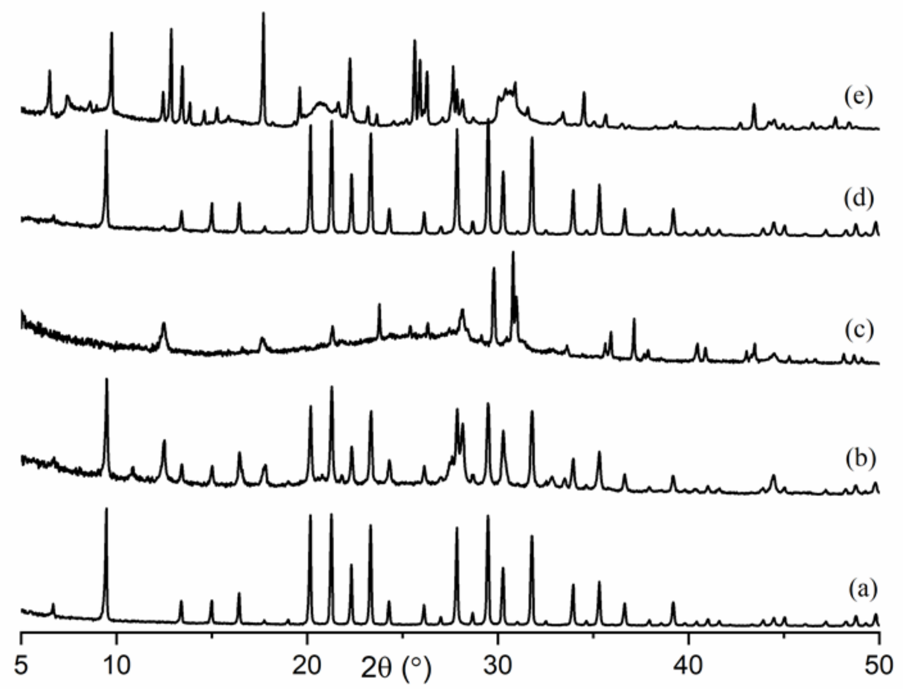

Figure S10. PXRD patterns of materials synthesised in presence of (a) KNO_3 + $NaNO_3$, (b) K_3PO_4 + $NaNO_3$, (c) K_3PO_4 + Na_2SO_4, (d) KNO_3 + Na_2SO_4 and (e) only $NaNO_3$ in starting gel.

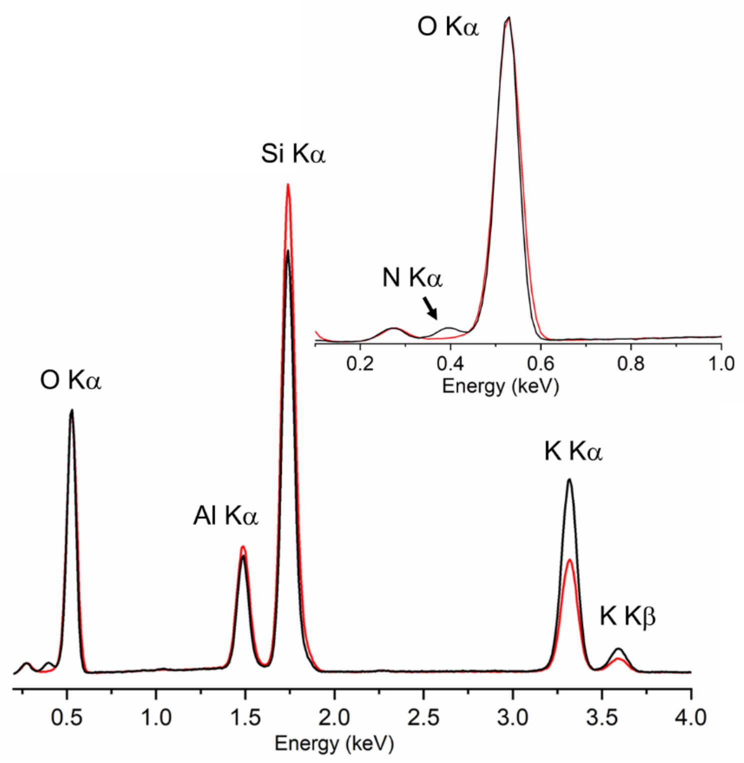

Figure S11. EDS analysis of K,NO_3-ZK-5 (black) and K-ZK-5(S) (red). Insert is showing a magnified view of N K_α peak at 0.392 keV.

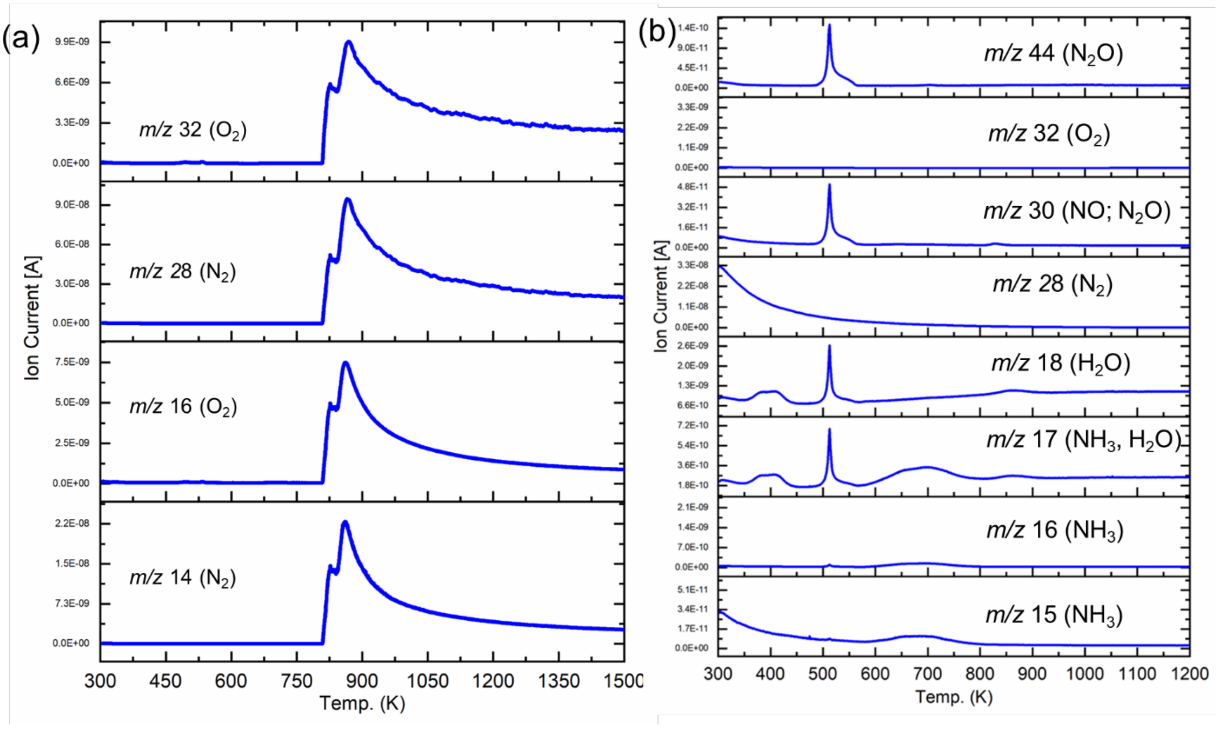

Figure S12. TG-MS in Ar of (a) K,NO_3-ZK-5 and (b) NH_4,NO_3-ZK-5 (heating rate 5 K min^{-1}).

Table S1. Crystallographic details of all samples. Shortcuts ‘deh’ and ‘hyd’ refer to dehydrated and hydrated samples, respectively.

	**K,NO$_3$-ZK-5 (deh)**	**K,Cs,NO$_3$-ZK-5 (deh)**	**K,Cs,NO$_3$-ZK-5 (hyd)**
**Unit cell**	$K_{33.1}(NO_3)_{17.9}[Al_{19.6}Si_{76.4}O_{192}]$	$K_{24.2}Cs_{11}(NO_3)_{17.7}[Al_{16.8}Si_{79.2}O_{192}]$	$K_{25.2}Cs_{10.8}(NO_3)_{22.1}[Al_{16.8}Si_{77.2}O_{192}]$
**Temperature/K**	298	298	298
**Space group**	$Im\bar{3}m$	$Im\bar{3}m$	$I4/mmm$
**X-ray source**	Mo	Mo	Cu
**Diffractometer**	Stoe	Stoe	Panalytical
**Wavelength (Å)**	0.70926	0.70926	1.54056
**a/ Å**<br>**b/ Å**<br>**c/ Å**	18.618(1)<br>18.618(1)<br>18.618(1)	18.604(1)<br>18.604(1)<br>18.604(1)	18.629(1)<br>18.629(1)<br>18.684(1)
**Volume/Å^3**	6454(1)	6439(1)	6484(1)
**R_p**	5.8%	5.6%	7.4%
**R_{wp}**	7.4%	7.1%	10.0%
**χ^2**	135	156	6

Table S2. Fractional atomic coordinates, occupancies, multiplicities and isotropic displacement parameters (in Å^2).

**K,NO$_3$-ZK-5 (deh)**	**Type**	**x**	**y**	**z**	**Occup.**	**Mult.**	**Biso**
Si1	Si	0.085(1)	0.202(1)	0.319(1)	0.81	96	1
Al1	Al	0.085(1)	0.202(1)	0.319(1)	0.19	96	1
O1	O	0.127(1)	0.127(1)	0.314(1)	1	48	1
O2	O	0.255(1)	0.255(1)	0.409(1)	1	48	1
O3	O	0	0.186(1)	0.337(1)	1	48	1
O4	O	0.25	0.113(1)	0.387(1)	1	48	1
K1	K	0.134(1)	0.134(1)	0.134(1)	0.81(1)	16	2
K2	K	0	0.25	0.5	0.82(1)	12	2
K3	K	0	0.338(1)	0	0.86(1)	12	2
N1	N	0	0.181(1)	0	0.93(1)	12	2
ON11	O	0	0.181(1)	0.067	0.23(1)	48	2
ON12	O	-0.058	0.181(1)	-0.034	0.12(1)	96	2
ON13	O	0.058	0.181(1)	-0.034	0.12(1)	96	2
N2	N	0.549(1)	0.451(1)	0	0.26(1)	24	2
ON21	O	0.527(2)	0.515(1)	0	0.13(1)	48	2
ON22	O	0.615(1)	0.438(2)	0	0.13(1)	48	2
**K,Cs,NO$_3$-ZK-5 (deh)**	**Type**	**x**	**y**	**z**	**Occup.**	**Mult.**	**Biso**
Si1	Si	0.084(1)	0.202(1)	0.320(1)	0.81	96	1
Al1	Al	0.084(1)	0.202(1)	0.320(1)	0.19	96	1
O1	O	0.126(1)	0.126(1)	0.314(1)	1	48	1
O2	O	0.257(1)	0.257(1)	0.408(1)	1	48	1
O3	O	0	0.184(1)	0.334(1)	1	48	1
O4	O	0.25	0.114(1)	0.386(1)	1	48	1
K1	K	0.132(1)	0.132(1)	0.132(1)	0.88(1)	16	2
K2	K	0	0.25	0.5	0.84(1)	12	2
Cs3	Cs	0	0.352(1)	0	0.91(1)	12	2
N1	N	0	0.180(1)	0	0.90(1)	12	2
ON11	O	0	0.180(1)	0.067	0.23(1)	48	2
ON12	O	-0.058	0.180(1)	-0.034	0.11(1)	96	2
ON13	O	0.058	0.180(1)	-0.034	0.11(1)	96	2

N2	N	0.572(1)	0.428(1)	0	0.28(1)	24	2
ON21	O	0.565(2)	0.494(1)	0	0.14(1)	48	2
ON22	O	0.634(1)	0.401(2)	0	0.14(1)	48	2
**K,Cs,NO_3-ZK-5 (hyd)**	**Type**	**x**	**y**	**z**	**Occup.**	**Mult.**	**Biso**
Si1	Si	0.084(1)	0.205(1)	0.320(1)	0.81	32	1
Al1	Al	0.084(1)	0.205(1)	0.320(1)	0.19	32	1
Si1a	Si	0.318(1)	0.083(1)	0.202(1)	0.81	32	1
Al1a	Al	0.318(1)	0.083(1)	0.202(1)	0.19	32	1
Si1b	Si	0.200(1)	0.321(1)	0.085(1)	0.81	32	1
Al1b	Al	0.200(1)	0.321(1)	0.085(1)	0.19	32	1
O1	O	0.126(1)	0.126(1)	0.312(1)	1	16	1
O2	O	0.259(1)	0.259(1)	0.411(1)	1	16	1
O3	O	0	0.182(2)	0.326(2)	1	16	1
O4	O	0.114(1)	0.386(1)	0.25	1	16	1
O1a	O	0.314(1)	0.124(1)	0.123(1)	1	32	1
O2a	O	0.407(1)	0.258(1)	0.258(1)	1	32	1
O3a	O	0.338(2)	0.185(2)	0	1	16	1
O3b	O	0.333(2)	0	0.185(1)	1	16	1
O4a	O	0.25	0.116(1)	0.386(1)	1	32	1
K1	K	0.131(1)	0.131(1)	0.131(1)	0.90(1)	16	2
K2	K	0	0.5	0.25	0.91(3)	4	2
K2a	K	0.5	0.25	0	0.92(2)	8	2
Cs3	Cs	0	0	0.356(1)	0.90(1)	4	2
Cs3a	Cs	0	0.354(1)	0	0.90(1)	8	2
N1	N	0	0.177(1)	0	0.96(2)	8	2
ON11	O	0	0.177(1)	0.067	0.48(1)	16	2
ON12	O	-0.058	0.177(1)	-0.033	0.24(1)	32	2
ON13	O	0.058	0.177(1)	-0.033	0.24(1)	32	2
N2	N	0.591(1)	0	0.409(1)	0.42(1)	16	2
ON21	O	0.526(1)	0	0.393(2)	0.42(1)	16	2
ON22	O	0.637(2)	0	0.361(2)	0.42(1)	16	2
ON23	O	0.609(2)	0	0.474(1)	0.42(1)	16	2
N1a	N	0	0	0.175(1)	1.00(3)	4	2
ON11a	O	0	-0.067	0.175(1)	0.25(1)	16	2
ON12a	O	-0.058	0.034	0.175(1)	0.13(1)	32	2
ON13a	O	0.058	0.034	0.175(1)	0.13(1)	32	2
N2a	N	0.577(2)	0.423(2)	0	0.47(2)	8	2
ON21a	O	0.527(3)	0.379(3)	0	0.23(1)	16	2
ON22a	O	0.563(3)	0.489(2)	0	0.23(1)	16	2
ON23a	O	0.640(2)	0.403(3)	0	0.23(1)	16	2

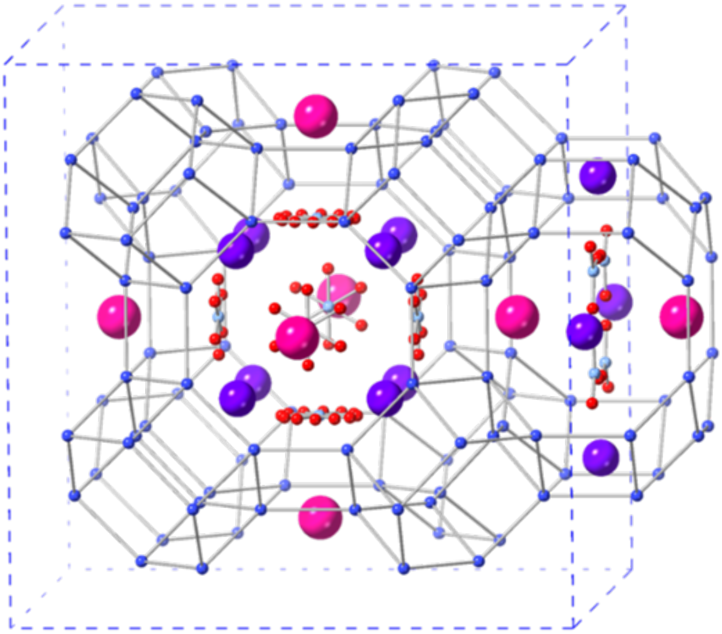

Figure S13. Generalised model of the structure of hydrated (*I*4/*mmm* symmetry) zeolite K,Cs,NO_3-ZK-5 obtained from Rietveld refinement (K = purple spheres, Cs = pink spheres, O = red spheres, T = dark blue spheres and N = pale blue spheres). Framework O atoms are omitted for clarity and T-T linkages are represented by grey rods.

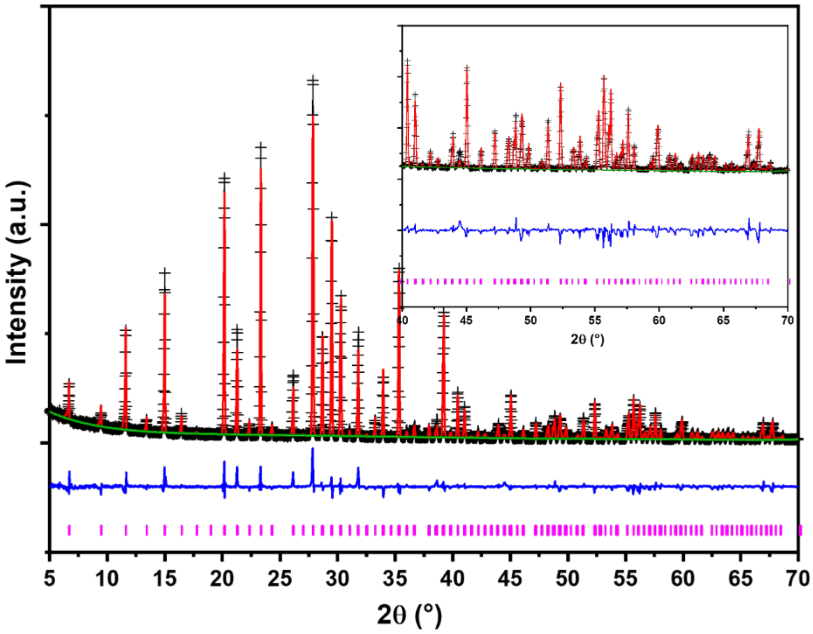

Figure S14. Rietveld plot of PXRD data (λ = 0.70926 Å, T = 298 K) of hydrated K,Cs,NO_3-ZK-5 (Observed – blue, calculated – red, difference – grey, phase – blue and background – grey).

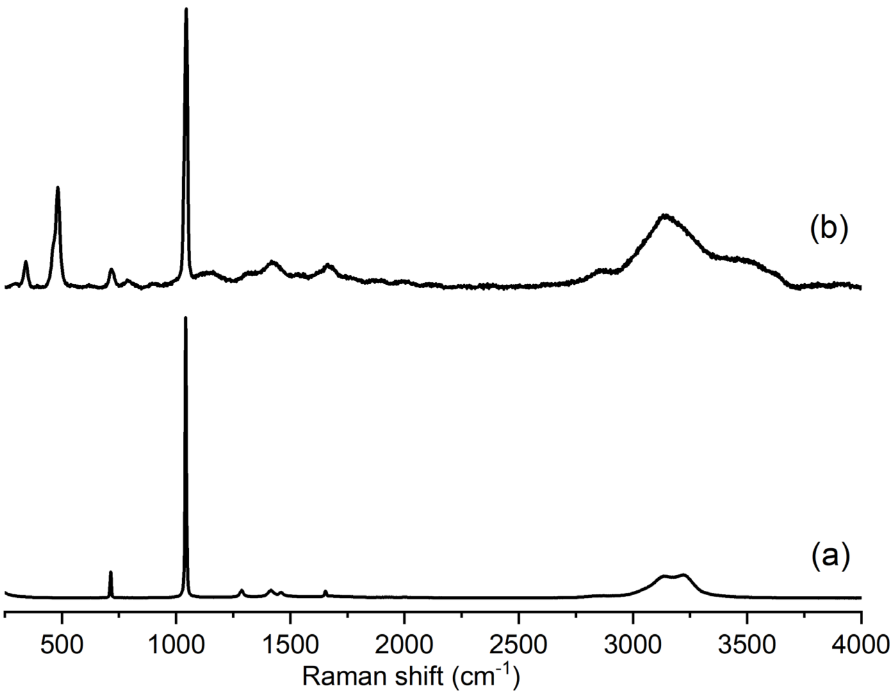

Figure S15. Raman spectra of (a) NH_4NO_3 and (b) NH_4,NO_3-ZK-5.

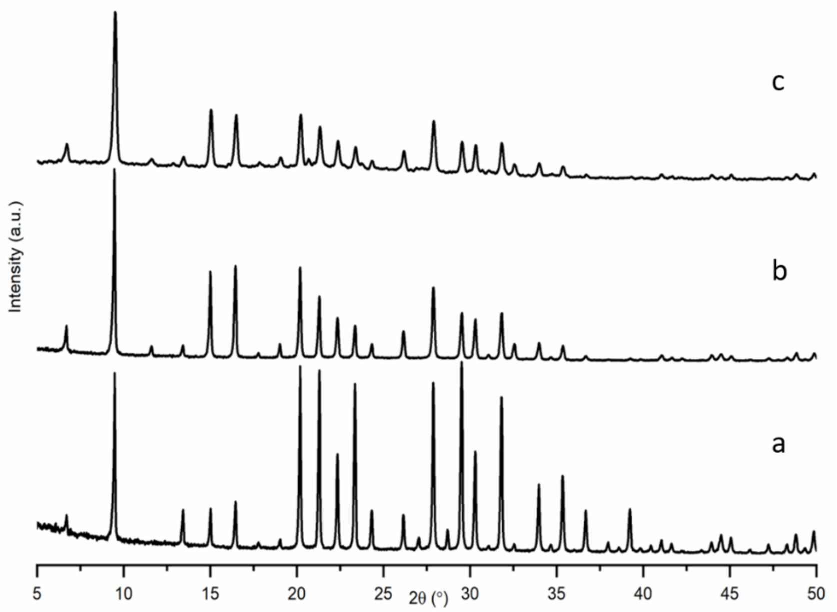

Figure S16. PXRD patterns of (a) NH_4,NO_3-ZK-5, (b) H-ZK-5 prepared by calcination of NH_4,NO_3-ZK-5 at 823 K and (c) H-ZK-5 prepared via synthesis route of Chatelain,[2] plus calcination, ammonium exchange and deammoniation.

S4. Experimental

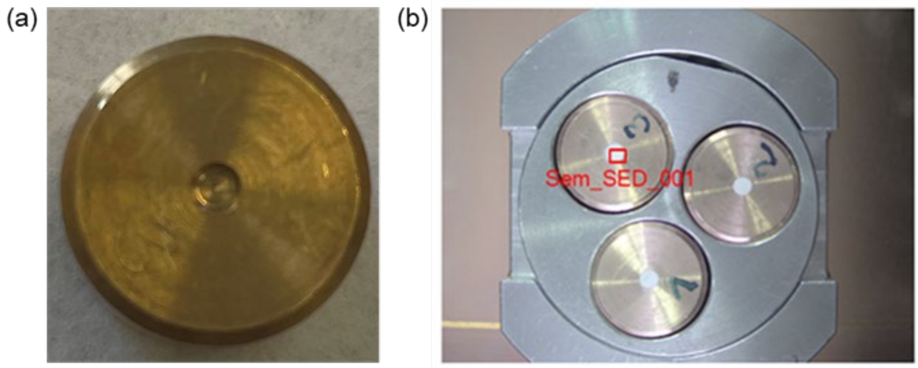

Figure S17. (a) Custom-made brass stub for EDS measurements. (b) Stubs with samples inside the SEM instrument.

References

[1] J. Kim, S. J. Cho, D. H. Kim, *ACS Catal.* **2017**, *7*, 6070–6081.

[2] T. Chatelain, J. Patarin, R. Farre, O. Petigny, P. Schulz, *Zeolites* **1996**, *17*, 328–333.

[3] IZA database http://www.iza-structure.org/databases/
